# Yellow fever vaccination coverage among nomadic populations in the Savannah Region, Ghana: a cross-sectional study following an outbreak

**DOI:** 10.11604/pamj.2026.53.24.49809

**Published:** 2026-01-16

**Authors:** Abdul-Wahab Inusah, Mohammed Mutaru Tahiru, Collins Gbeti, Peter Dzomeku, Michael Head, Shamsu-Deen Ziblim

**Affiliations:** 1 Department of Global and International Health, School of Public Health, University for Development Studies, Tamale, Ghana,; 2 Northern Regional Health Directorate, Ghana Health Service, Tamale, Ghana,; 3 Drylands Research Institute, University for Development Studies, Tamale, Ghana,; 4 Department of Internal Medicine, Tamale Teaching Hospital, Tamale, Ghana,; 5 Clinical Informatics Research Unit, Faculty of Medicine, University of Southampton, Southampton, United Kingdom,; 6 Directorate of Academic Planning and Quality Assurance, University for Development Studies, Tamale, Ghana

**Keywords:** Yellow fever vaccination, nomadic populations, vaccination coverage, Ghana, herd immunity

## Abstract

**Introduction:**

Yellow fever (YF) remains a major public health concern in Ghana, with periodic outbreaks despite ongoing vaccination efforts. Nomadic populations, due to their mobility and remote settlements, are often underserved by vaccination campaigns, posing challenges to achieving herd immunity. The objective of the study was to estimate yellow fever vaccination coverage among nomadic populations in the Savannah Region of Ghana and compare it with the national average, and identify reasons for non-vaccination to inform future outbreak response strategies.

**Methods:**

a community-based cross-sectional study was conducted among 2,914 individuals from 414 nomadic households across 22 affected communities using a modified WHO vaccination coverage survey. Data were analyzed in Stata version 15. Descriptive statistics and t-tests were used to assess differences in vaccination coverage and associated factors.

**Results:**

overall vaccination coverage was 80.3% (SD = 0.24), significantly lower than the national average of 88% (t(413) = -4.00, p < 0.001), though within the WHO-recommended threshold for herd immunity. A significant inverse relationship was observed between household size and vaccination coverage (p < 0.001). Most respondents (93.2%) presented vaccination cards for verification, while 4.8% reported verbally. The main reasons for non-vaccination included absence during campaigns, lack of transportation, and limited information about the campaign. Perceptions of vaccine effectiveness were largely positive (67.5%), though 25.1% expressed doubts about efficacy.

**Conclusion:**

yellow fever vaccination coverage among nomadic populations in the Savannah Region, though adequate for herd protection, remains below national targets. Strengthened outreach strategies, tailored health promotion, and targeted catch-up campaigns are essential to sustain high coverage and prevent future outbreaks among mobile and hard-to-reach populations.

## Introduction

Yellow fever (YF) is a flavivirus-borne acute systemic illness spread by infected mosquitoes of the *Aedes* and *Haemagogus* species [1]. YF is difficult to diagnose since *the* symptoms and signs are similar to those of other diseases such as malaria, typhoid, dengue fever, and other haemorrhagic fevers [2]. The disease can be transmitted to both human and non-human primates through *Aedes* mosquitoes spp in Africa and *Haemagogus* spp and *Sabethes* spp in Southern America [3]. Yellow fever has three cycles of transmission: the sylvatic cycle, which involves non-human primates (*Aedes* mosquitoes to monkeys found in the forest). The intermediate cycle involves non-human primates, humans and *Aedes* spp. mosquitoes in African savannah settings, whilst the third cycle is the urban cycle involving *Aedes aegypti* mosquitoes and humans in cities. However, the sylvatic and the intermediate cycles are the most common means of YF transmission in many high-endemic African States [2]. There are international targets as part of the 'Eliminate Yellow Fever Epidemics (EYE)' initiative, with the primary outcome being to end YF epidemics by 2026 [4]. Annually, as of 2020, 200,000 cases of YF are reported in Africa and South America, with 90% of these cases occurring in Africa, resulting in an estimated 30,000 deaths [5]. By region, West and Central Africa are reported to have the highest cases, with about 300 probable and 88 laboratory-confirmed cases since 2021 [6], with frequent outbreaks being considered a local and global health threat [7]. A majority of yellow fever cases in Africa are found among unvaccinated people living in the YF endemic zones [5,8]. There is no known treatment or medication for YF. However, vaccines have been a very effective tool in the prevention of YF, with protection typically lasting for many years in over 80% of vaccinated individuals [9].

The estimated threshold for herd immunity and thus reduction in numbers and extent of the outbreak is thought to be around 80% [6]. As of 2020, Africa's YF vaccination coverage was 44%, with the WHO citing this as one of the key challenges in controlling the incidence of YF [6]. National coverage varies across West and Central Africa, for example, Ghana (88%), Côte d'Ivoire (69%), Cameroon (57%), Democratic Republic of Congo (DRC, 56%), Nigeria (54%), Central African Republic (CAR, 41%), Chad (35%) and Angola (30%) [10]. Overall, this often low vaccination coverage makes the population susceptible to YF and the possible re-emergence of YF in these countries [11,12]. Even though national YF vaccination coverage in Ghana is relatively high [10], the country has seen a re-emergence of the disease. Vaccination is not mandatory for Ghanaian residents, unlike travellers who arrive at ports of entry into the country. From October 15 to 27^th^ November 2021, 202 suspected cases of YF, including 70 confirmed positive cases with 35 deaths (17% case fatality ratio), were reported in four regions of Ghana (Upper West, Savannah, Bono, and Oti regions) [13]. A vast majority of the positive cases reported were among the nomadic population suspected to be migrating from Nigeria into the Ghana Savannah forest reserves through its porous borders [13]. The Ghana Health Service and partners, since November 2021, have conducted focused YF vaccination campaigns in 80 targeted communities in the North Gonja and West Gonja Districts of the Savannah region. Efforts are also made to continue the catch-up vaccination campaigns using the routine immunisation system. Members of the nomadic communities initially reported symptoms to their local health centre, where YF was suspected and then confirmed. An immunisation campaign was approved at the municipal (district) and regional levels. The Ghana Health Service team, including the use of residents as community health volunteers (CHVs), transported the YF vaccines into the field each day in vaccine carriers ('cool bags'). Unused vaccines are returned for storage at a designated health facility with a cold chain. There was a previous YF outbreak among nomadic communities in 2019. The resident population at that time, many of whom received a vaccination, had previously moved on with their livestock to new pastures. This study is part of ongoing efforts to understand YF prevention and control among at-risk populations in Ghana, particularly nomadic communities [14]. Given the vulnerability observed in nomadic groups during recent outbreaks in the Savannah region, this research estimates the prevalence of YF vaccination coverage among nomadic populations residing in affected communities of the West Gonja district. Specifically, the study aims to determine the proportion of nomadic individuals vaccinated following focused vaccination campaigns. Recognising that Ghana's national YF vaccination coverage is approximately 88%, this study also evaluates whether vaccination rates within these nomadic populations differ significantly from this national average. Additionally, the study investigates the reasons for non-vaccination among these populations. Understanding these barriers is critical for informing health authorities and tailoring future vaccination campaigns to improve reach among mobile and hard-to-reach populations.

## Methods

**Study area, design, and period:** a community-based cross-sectional survey was conducted between February and March 2022 among nomadic households in 22 communities affected by a YF outbreak in the West Gonja Municipal, Savannah Region, Ghana. The vaccination campaign in these communities was completed in November 2021. West Gonja Municipal, with a population of 63,449, is one of seven administrative districts in the Savannah Region and serves as the regional administrative capital. It borders Côte d'Ivoire and Burkina Faso and covers a landmass of 4,715.9 square kilometres, including part of the protected Mole National Park [14,15].

**Study population, participants, and inclusion criteria:** the study population comprised all members of nomadic households residing in the outbreak-affected communities. For this study, nomads were defined as people without fixed habitation who regularly move in search of greener pastures for livestock, farmland, or both. Excluded were non-nomadic households and individuals ineligible for the yellow fever vaccine, specifically, children under 9 months, pregnant women, and persons allergic to egg products [16]. A household was defined as a single building or structure housing one family unit. Household heads or spouses present and able to provide informed consent were interviewed. When household members were absent, information was provided by the head or spouse respondent.

**Sampling frame and technique:** communities were purposively selected based on the yellow fever case line list provided by the Municipal Assembly Health Directorate, which detailed confirmed cases during the outbreak. In each selected community, CHVs who had participated in the recent YF vaccination campaign assisted as focal persons to identify nomadic households. Physical marks on the household structure, indicating evidence of vaccinated household members, complemented the work of the CHVs. A snowball sampling approach was employed, whereby consented household heads or spouses were asked to identify additional nomadic households for inclusion.

**Sample size calculation:** a sample size of 377 nomadic households was calculated to achieve study objectives at a 95% confidence interval, assuming 50% vaccination coverage, a margin of error of 5%, and inflation by 5% for anticipated nonresponse or incomplete data.

**Data collection tool and procedure:** data collection was conducted using a structured questionnaire adapted from the WHO post-vaccination cluster survey form [17] and previous similar studies. The questionnaire comprised two primary sections:

***Section 1:*** demographic characteristics including gender, age, marital status, religious affiliation, household size, duration of stay, intention to relocate, and nationality.

***Section 2:*** YF vaccination coverage information, with vaccination status verified using vaccination cards signed by the Ghana Health Service or verbal confirmation for missing/absent members.

Questionnaires were uploaded onto the Android Open Data Kit (ODK) mobile application and pretested in the North Gonja District among a comparable population. Trained research officers conducted face-to-face interviews using local dialects, predominantly Hausa, Fulani, Dagbani, and Gonja, due to low literacy levels in the study population. Each household interview lasted approximately 25 minutes. Five research officers collected data over a period of seven days.

**Data analysis:** data were cleaned in Microsoft Excel 2019 and analysed using Stata version 15. Descriptive statistics, summarised demographic and vaccination coverage information were presented in tables and charts. Further analyses included: A paired sample t-test to assess the difference between household size and household vaccination coverage. A one-sample t-test comparing the household vaccination coverage in the study population with the national YF vaccination coverage of 88%. An independent sample t-test comparing vaccination coverage between native and foreign nomadic groups. All statistical tests were conducted after verifying assumptions, including normality.

**Ethical considerations:** ethical approval was obtained from the University for Development Studies (UDS) Research and Ethics Review Board (approval number: UDS/RB/013/22). Permissions were also granted by the Savannah Regional Health Directorate. Study objectives were explained to participants, and confidentiality and privacy were maintained throughout. Participation was voluntary, with informed consent obtained via signature or thumbprint before data collection. At the end of each interview, research officers provided five minutes of health education to households on YF signs, symptoms, prevention strategies, and the benefits.

## Results

**Socio-demographic characteristics of the study population:** a total of 414 nomadic households participated in the survey. Among the household heads, 57.7% were male, with a mean age of 38.5 years (±13.1), ranging from 18 to 84 years. Most participants were married (91%). The majority of respondents were herdsmen by occupation (67.4%). By nationality, 56% were foreign nomads, predominantly migrating from the Benin Republic (Table 1).

**Table 1:** socio-demographic characteristics of nomadic households (N=414) surveyed in the West Gonja Municipal, Savannah region, Ghana, 2022

Variable	Frequency N=414	Percentage%
**Age group**	**Mean 38.5 ± 13.1**	
18-34	187	45.20
35-51	152	36.70
52-68	64	15.50
69-85	11	2.70
**Household size**	**Mean 7.04 ± 3.7**	
1 to 5 inclusive	164	39.60
6 to 10 inclusive	189	45.70
>11+	61	14.70
**Gender**		
Female	175	42.30
Male	239	57.70
**Marital status**		
Never married	21	5.10
Married	375	90.60
Widowed	18	4.40
**Main occupation**		
Agro-pastoralist (mix farming)	114	27.50
Herdsman	279	67.40
Trader/Vendor	21	5.10
**Religious affiliation**		
Islam	414	100
**Nationality**		
Ghanaian	184	44.40
Foreigner	230	55.60
**Foreign country**		
Benin	119	51.70
Burkina Faso	36	15.70
Nigeria	55	23.90
Togo	20	8.70

**Prevalence of yellow fever vaccination coverage among nomadic populations:** a total of 2,914 household members from the surveyed nomadic households were included in the study. Of these, 2,342 individuals reported having ever received the yellow fever vaccine, resulting in an overall vaccination coverage of 80% following the outbreak response and vaccination campaigns. Vaccination verification showed that 2,156 (92%) of the vaccinated individuals presented official yellow fever vaccination cards as evidence. The remaining 8% either reported losing their vaccination cards or were unable to produce them at the time of data collection (Table 2).

**Table 2:** yellow fever vaccination status among household members (N=2,914) in nomadic communities of West Gonja Municipal, Savannah Region, Ghana, 2022

Totals	Members	Average members	Difference	Vaccination coverage
Household members	2,914	7.04	572	80%
Members vaccinated	2,342	5.7		

**Vaccination coverage compared to national estimates (80%):** a one-sample t-test was performed to compare yellow fever vaccination coverage among household members in the study population to the national vaccination coverage rate of 88%. The mean vaccination coverage in the nomadic population was approximately 80.3% (SD = 0.24), which was significantly lower than the national average (t(413) = -4.00, p < 0.001). This 7.7% difference underscores a coverage gap in this vulnerable group. Further analysis using paired sample t-tests indicated a negative association between household size and vaccination coverage; larger households exhibited lower vaccination rates per member, suggesting household size as a barrier to achieving optimal vaccination within nomadic communities. In contrast, an independent samples t-test showed no significant difference in vaccination coverage between native and foreign nomadic groups (p = 0.31), indicating that nomadic origin did not significantly influence vaccine uptake (Table 3, Table 4).

**Table 3:** comparison of yellow fever vaccination coverage among nomadic populations in West Gonja Municipal, Ghana, to national coverage, and vaccination associations with household size and nomadic origin, 2022

items	Population	Obs	Members	Mean	SD	SE	t	P-Value	95%(CL)
Group-A	Study population Vs National Coverage (88%)	414	2342	0.80	0.24	0.01	-4.00	0.001	0.81-0.86
Group-B	Household size	414	2914	7.04	3.68	0.18	11.83	0.001	6.68-7.39
	Household Vaccination Coverage	414	2342	5.66	3.13	0.15			5.36-5.96
Group-C	Native nomadic	184	1088	5.44	3.87	3.87	1.023	0.31	4.88-6.00
	Foreign nomadic	230	1254	5.087	2.43	2.43			4.33-5.44

Obs = Observations; SD = Standard Deviation; SE = Standard Error. Group A represents a one-sample t-test comparing study vaccination coverage against the national reported coverage. Group B presents a paired sample t-test for household size versus household vaccination coverage. Group C shows an independent samples t-test comparing vaccination coverage between native and foreign nomadic groups.

**Table 4:** yellow fever vaccination status and perceptions among household heads (N=414) in nomadic communities, West Gonja Municipal, Ghana, 2022

Variable	Category	Frequency	Percentage (%)
Have you ever been vaccinated against YF?	No	8	1.93
	Yes, cards not seen	20	4.83
	Yes, cards seen	386	93.24
When did you receive your first YF vaccination?	Before the most recent outbreak	7	1.72
	During the just-ended campaign	399	98.28
How effective do you think the vaccine is in preventing yellow fever?	Not effective at all	102	25.12
	Slightly effective	19	4.68
	Moderately effective	11	2.71
	Very effective	175	43.1
	Extremely effective	99	24.38

**Figure 1:**
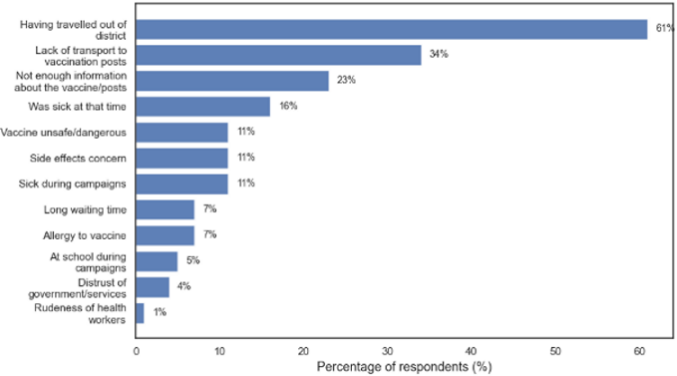
reported reasons for non-vaccination among household member s(N=572) in nomadic communities, West Gonja Municipal, Ghana, 2022 (this was a multiple response question)

**Reasons for non-vaccination among household members:** of the 2,914 household members included in the study, 572 were not vaccinated against YF. Several reasons for non-vaccination were reported. The most common reason, cited by 61%, was that individuals had travelled out of the district during the vaccination campaign period, making it impossible for them to receive the vaccine. Lack of transportation to vaccination posts was the second most frequent barrier, reported by 34% of respondents. Other reasons included insufficient information about the vaccine or vaccination sites (23%), illness at the time of the campaign (16%), and concerns about vaccine safety or potential side effects (each reported by 11%). Additionally, 11% of participants were sick during the vaccination campaigns, 7% expressed fear of allergic reactions, and 7% reported long waiting times at vaccination posts. Less frequently cited barriers included being at school during the campaigns (5%), distrust of government or health services (4%), and experiencing rudeness from health workers (1%) (Figure 1).

**Household heads' yellow fever vaccination status and perceptions (Supplementary data):** in addition to the primary study objectives, supplementary data were collected on household heads' vaccination status and perceptions to provide further context. Among 414 household heads interviewed, only 8 (1.93%) reported never receiving a YF vaccination, although other household members had been vaccinated. The majority (93.24%) provided vaccination cards as proof, and 4.82% used verbal reports due to missing cards. Most participants (98.3%) received their first vaccination during the most recent vaccination campaign. A higher proportion of foreign nomadic household heads (60%) were vaccinated compared to local nomadic heads (40%); however, no significant difference was found in overall coverage between these groups. When asked about vaccine effectiveness, 67.5% perceived it as effective or very effective, while 25.1% felt it was not effective.

## Discussion

This study was conducted in the West Gonja Municipality after a focused vaccination campaign carried out in over 80 communities of the West Gonja and North Gonja Districts of the Savannah Region. The primary objectives of this study were to estimate the prevalence of YF vaccination coverage among nomadic populations residing in affected communities, to compare this coverage with Ghana's national average of 88% [13], and to investigate reasons for non-vaccination in this population.

Our study found YF vaccination coverage to be 80% among the study population. Although this finding was below the national vaccination coverage of 88% [13], it was still within the WHO recommended threshold for attaining herd immunity [13,17]. A one-sample t-test comparing the nomadic population's mean vaccination coverage of 80.3% (SD = 0.24) against the national average of 88% found the difference to be statistically significant (t(413) = -4.00, p < 0.001), indicating a meaningful coverage gap in this vulnerable group. Several factors likely contribute to this lower coverage. Nomadic populations are often highly mobile and settle in remote, forested, or hard-to-reach areas, which limits consistent access to vaccination services. Their migratory lifestyle hinders completion of multi-dose vaccine schedules due to loss to follow-up, compounded by limited health infrastructure in these regions [18,19]. Additionally, challenges such as language barriers, cultural perceptions, and occasional discrimination by healthcare providers further impede vaccine uptake among nomadic groups [20-22]. Tailored, community-engaged interventions, including involving local leaders and integrating health services, have shown promise in improving vaccination coverage in these populations [18,21].

The coverage observed in this study likely resulted from the compulsory nature of vaccination among travellers and the proactive approach towards community vaccination in response to the recent YF outbreak within the Savannah region [13]. Additional opportunities for catch-up vaccinations outside the immediate campaign may also have contributed. However, communities outside the vaccination campaign might have lower coverage due to the population's scattered, highly mobile, and remote settlements. On the contrary, patterns of lower vaccination coverage among nomadic populations were reported in Kenya, with vaccination rates less than 25% attributed to their mobility and limited access to health services [18]. As highlighted by Ghana's national efforts, routine vaccinations have the potential to reach much of the target population. Another example is in French Guiana, where national vaccination coverage is reported at 95% [12].

The findings revealed no statistically significant difference in vaccination coverage between local nomadic populations and international nomadic groups. An independent samples t-test confirmed the absence of a significant effect of nomadic origin on vaccine uptake (p = 0.31), suggesting that the vaccination campaign equally reached diverse nomadic subgroups. This equitable coverage likely reflects the implementation of a house-to-house vaccination strategy, wherein each household was systematically visited, thereby ensuring uniform access to vaccination services. The campaign's design emphasised free vaccine delivery directly within communities and provided multiple opportunities for vaccination, effectively mitigating common barriers such as financial cost, opportunity costs, and physical access to healthcare facilities [18,22].

Further, paired sample t-tests indicated a significant inverse relationship between household size and vaccination coverage, suggesting that larger households experienced lower vaccination rates per member. Given that WHO currently recommends a single dose of the YF vaccine to confer lifelong immunity, which has been incorporated into the Expanded Program on Immunization (EPI) for children aged 9-12 months in endemic countries like Ghana [23], this association may stem from challenges such as vaccine hesitancy among household members or increased mobility leading to absenteeism during vaccination rounds [13,24]. These findings implicate incomplete household vaccination as a potential risk factor for sustained transmission and future outbreaks. Consequently, public health authorities should consider targeted catch-up vaccination campaigns in communities identified as having suboptimal coverage to achieve and maintain the national vaccination threshold [25]. Moreover, updated population and census data with accurate estimates of nomadic household sizes are critical for effective health service planning and logistical optimisation.

The study examined the reasons for non-vaccination to inform more efficient planning for future outbreak control. Consistent with prior work, the key barriers cited were being out of district during campaigns, lack of transport to vaccination posts, and limited information about the campaign. Similar access challenges and information gaps have been documented in Ghanaian settings, such as among nomadic populations [22] and among people with chronic conditions [26]. In broader sub-Saharan Africa, insufficient awareness, beliefs about vaccine harm, and health system constraints have been repeatedly observed [25,27]. In Malawi, for example, equity and access issues emerge as a significant factor shaping vaccine uptake [28]. Moreover, institutional trust and community engagement play critical roles: in multi-country analyses, trust in government and society was inversely associated with vaccine hesitancy [29]. These findings underscore the imperative for sustained motivation among health workers to reach remote communities, enhanced logistics, coordinated planning, and tailored health promotion to reduce misinformation and improve uptake.

The supplementary data reveal a very high vaccination coverage among household heads, with only 1.93% unvaccinated. Most respondents (93.24%) presented vaccination cards, indicating reliable reporting and effective documentation. Nearly all (98.3%) received their vaccination during the most recent campaign, underscoring the success of outreach efforts in reaching even mobile and nomadic groups. Although foreign nomadic heads had slightly higher coverage (60%) than local nomadic heads (40%), the difference was not statistically significant, suggesting equitable campaign access across groups. Perceptions of vaccine effectiveness were largely positive, with 67.5% of respondents considering the vaccine effective or very effective. However, about one-quarter (25.1%) perceived it as ineffective, highlighting lingering misconceptions that could impede future uptake. Similar findings have been reported in Ghana and across sub-Saharan Africa, where misinformation, perceived inefficacy, and limited trust in health systems have been linked to vaccine hesitancy [25,27]. Addressing these barriers requires sustained community engagement, culturally appropriate health communication, and strengthened trust between healthcare providers and mobile populations to maintain high coverage and prevent future YF outbreaks.

**Strengths and limitations:** this study has several strengths and limitations that warrant consideration. A key strength lies in its focus on a nomadic population, a group often underrepresented in vaccination coverage studies despite their increased vulnerability to infectious disease outbreaks. The use of household heads as respondents provided comprehensive household-level information, ensuring accurate reporting of demographic characteristics and vaccination status across members. Additionally, data collection occurred immediately after the YF vaccination campaign, minimising recall bias and enhancing the reliability of responses. The high proportion of respondents who could provide vaccination cards (over 93%) further supports the validity of the reported vaccination coverage.

However, the study is not without limitations. The use of proxy interviews may have introduced information bias, as responses were based on the household head's account rather than direct individual verification. Selection bias is also a potential concern, though likely minimal due to the small, close-knit nature of the nomadic communities, where members have substantial knowledge of each other. Recall bias may have affected participants who verbally reported vaccination status due to missing cards; however, this limitation likely had minimal influence given that only about 5% of respondents lacked card verification. Finally, the cross-sectional design restricts the ability to infer causality between socio-demographic factors and vaccination outcomes.

## Conclusion

This study assessed YF vaccination coverage among nomadic populations in the West Gonja Municipal following a focused vaccination campaign. The results showed that coverage (80%) among the nomadic population was below Ghana's national average of 88%, though still within the WHO-recommended herd immunity threshold. The coverage gap highlights the persistent challenges associated with mobility, remoteness, and access to health services among nomadic groups. No significant difference in vaccination coverage was found between local and foreign nomadic households, indicating equitable reach of the vaccination campaign. However, larger households were less likely to achieve full vaccination coverage, suggesting intra-household disparities. The main barriers to vaccination included absence during campaigns, transport constraints, and limited information about vaccination activities. Although most household heads viewed the vaccine as effective, a minority expressed doubts about its efficacy. These findings underscore the need for targeted catch-up vaccination campaigns in low-coverage areas, improved outreach logistics, and sustained community engagement to address information gaps and misconceptions. Strengthening trust and communication between health workers and nomadic populations will be critical to maintaining high coverage and preventing future outbreaks.

### What is known about this topic

- Nomadic and mobile populations are often underrepresented in vaccination programs due to their mobility and remote settlements;
- Access barriers and limited health communication remain key determinants of low vaccine uptake in rural and hard-to-reach communities;
- Misconceptions about vaccine safety and effectiveness contribute to hesitancy and suboptimal coverage.

### What this study adds

- This study provides empirical evidence of 80% yellow fever vaccination coverage among nomadic populations, below the national average but within WHO's herd immunity threshold;
- It shows that vaccination campaigns achieved equitable coverage among local and foreign nomadic subgroups through community-based outreach;
- It also identifies household size, absence during campaigns, transport barriers, and misinformation as persisting obstacles to full vaccination coverage.
